# Ablative Radiotherapy as a Strategy to Overcome TKI Resistance in EGFR-Mutated NSCLC

**DOI:** 10.3390/cancers14163983

**Published:** 2022-08-18

**Authors:** Jennifer Novak, Ravi Salgia, Howard West, Miguel A Villalona-Calero, Sagus Sampath, Terence Williams, Victoria Villaflor, Erminia Massarelli, Ranjan Pathak, Marianna Koczywas, Brittney Chau, Arya Amini

**Affiliations:** 1 Department of Radiation Oncology, City of Hope National Medical Center, Duarte, CA 91010, USA; 2 Department of Medical Oncology, City of Hope National Medical Center, Duarte, CA 91010, USA

**Keywords:** lung cancer, epidermal growth factor receptor (EGFR), EGFR-mutant, non-small cell lung cancer (NSCLC), stereotactic body radiation therapy (SBRT), stereotactic radiosurgery (SRS), resistance

## Abstract

**Simple Summary:**

Most patients with EGFR-mutated NSCLC who receive treatment with targeted therapy will eventually develop resistance, meaning the therapy will lose its efficacy. Prior studies have shown a benefit to continuing to treat patients on TKI therapy despite limited progression of one or more sites of metastatic disease in EGFR-mutated NSCLC. Based on the data reviewed here, the use of radiation therapy to sites of disease progression is both efficacious and carries a low risk for side effects, with the added benefit of allowing patients to continue on TKI therapy.

**Abstract:**

Tyrosine kinase inhibitor (TKI) therapy is the recommended first-line treatment for metastatic non-small-cell lung cancer (NSCLC) positive for epidermal growth factor receptor (EGFR) gene mutation. However, most individuals treated with TKI therapy for EGFR-mutant NSCLC will develop tumor resistance to TKI therapy. Therapeutic strategies to overcome TKI resistance are the topic of several ongoing clinical trials. One potential strategy, which has been explored in numerous trials, is the treatment of progressive sites of disease with stereotactic body radiation treatment (SBRT) or stereotactic radiosurgery (SRS). We sought to review the literature pertaining to the use of local ablative radiation therapy in the setting of acquired resistance to TKI therapy and to discuss stereotactic radiation therapy as a strategy to overcome TKI resistance.

## 1. Introduction

Lung cancer is the most common cancer worldwide (excluding skin cancers) and is also responsible for the highest global cancer-related mortality rates [1]. In 2021, there will be an estimated 235,760 new cases of lung cancer diagnosed and an estimated 131,880 deaths from lung cancer in the United States [2].

Lung cancer is divided into two main subgroups: small-cell lung cancer (SCLC) and non-small-cell lung cancer (NSCLC). Approximately 85% of lung cancers are classified as NSCLC, of which several genetic variants have been identified. The epidermal growth factor receptor (EGFR) oncogene is mutated in approximately 40–45% of NSCLCs [3,4,5]. Other commonly identified genetic mutations in NSCLC include rearrangement of the anaplastic lymphoma kinase (ALK) oncogene (present in up to 5% of NSCLCs), the Kirsten rat sarcoma viral oncogene homolog (KRAS) mutation (present in 20–30% of NSCLCs), and the tumor protein p53 (TP53) gene (up to 60% of cases) [6,7]. Over the past 20 years, targetable genetic mutations have been a primary focus among developers of novel anti-neoplastic therapies for NSCLC [8].

Most patients with EGFR-mutated NSCLC who receive treatment with targeted therapy will develop resistance. Despite advances in TKI development over the past two decades, acquired resistance remains a clinical problem even for the most modern TKIs. Strategies to overcome acquired resistance to targeted therapy are needed to improve oncologic outcomes for patients. In this review, we will discuss the relevant data and outline strategies for overcoming tumor resistance to targeted therapy using stereotactic radiation therapy.

## 2. Methods

We conducted a literature search using the MEDLINE database for articles meeting our search criteria published from January 2010 to March 2022 (Supplementary File S1). The following combination of keywords were searched: lung neoplasms, lung cancer, lung carcinoma, non-small cell lung cancer, NSCLC, metastatic, Stage IV or Stage 4, oligometastasis, oligometastases, oligometastatic, oligopersistent, oligoprogression, oligoprogressive, oligorecurrent, polymetastatic, external beam radiation therapy or external beam radiotherapy, stereotactic body radiation therapy or stereotactic body radiotherapy, SBRT, stereotactic ablative radiation therapy or stereotactic ablative radiotherapy, SABR, stereotactic radiosurgery, SRS, radiotherapy, chemotherapy, targeted therapy, epidermal growth factor receptor, EGFR, EGFR-mutant, resistant, refractory, randomized, and prospective. Reference lists were also reviewed of published manuscripts initially identified using the above search criteria with additional publications included.

## 3. Acquired TKI Resistance

The EGFR gene codes for a transmembrane receptor protein that is present on all cell types and plays several key roles in developmental, metabolic, and inflammatory cellular processes [9]. The EGFR receptor, also referred to as HER1, is one of several receptors in the HER receptor family. The extracellular component of the receptor is able to bind to several different ligands including epidermal growth factor (EGF), transforming growth factor alpha (TGFα), and amphiregulin, among others [10]. Upon binding an extracellular ligand, the EGFR receptor dimerizes and stimulates the receptor’s intracellular tyrosine kinase activity. The receptor then undergoes adenosine triphosphate (ATP)-dependent autophosphorylation and subsequent activation of downstream molecular signaling pathways such as the mitogen activated protein kinase (MAPK), phosphatidylinositol 3-kinase (PI3K), and janus kinase/signal transducers and activators of transcription (JAK/STAT) pathways [11].

The EGFR gene, located on the short arm of chromosome 7, is commonly mutated in tumor cells of various cancers, though these mutations appear to be most prevalent in NSCLC [12]. EGFR gene mutations typically result in overexpression or constitutive tyrosine kinase activity, leading to enhanced cancer growth. A vast majority of EGFR mutations in NSCLC are known as activating mutations and can result either from deletions or point mutations located within the tyrosine kinase domain on exons 18–21. Approximately 60% of EGFR mutations occur in exon 19 [13]. These mutations result in constitutive activation of the EGFR receptor’s tyrosine kinase activity. The incidence of EGFR mutations in NSCLC varies by population and has been reported in the literature to range anywhere from 15–50% [14,15]. Populations that are most likely to harbor EGFR mutations in NSCLC include females, East Asians, never-smokers, and those with tumors of adenocarcinoma histology [16].

EGFR tyrosine kinase inhibitors (TKIs) are a class of targeted drug therapies that competitively inhibit the binding of ATP, thereby preventing activation of tumorigenic molecular signaling cascades. The presence of EGFR mutations in NSCLC is both predictive and prognostic; not only do these mutations predict for clinical response to TKIs, but they also are associated with improved oncologic outcomes compared to non-EGFR mutated NSCLC [17]. Treatment of EGFR-mutated NSCLC with TKIs has been demonstrated to improve both progression free survival and overall survival [18,19,20]. TKIs can also directly mediate apoptosis [21]. Unfortunately, development of tumor cell resistance to TKI therapy typically occurs within 9–15 months after initiation of therapy [22]. Although there have been significant advancements in TKI therapies, development of tumor resistance to TKIs remains a clinical dilemma. Osimertinib is the most well-studied third generation TKI; however, the acquired C797S mutation represents the most common cause of resistance to Osimertinib [23].

Acquired resistance to TKI therapy is multifactorial and due in part to secondary EGFR gene mutations and the overexpression of various receptors and growth factors that modulate downstream signaling pathways. Secondary EGFR mutations commonly arise in individuals that have received TKI therapy [24]. One example of a secondary EGFR mutation is the T790M mutation, which enhances the binding of ATP and promotes activation of downstream signaling pathways. It is estimated that the T790M mutation is responsible for TKI resistance in 60% of patients receiving EGFR TKI therapy [25]. Studies have shown that secondary EGFR mutations are rare in NSCLC tumor cells prior to treatment with TKI therapies, suggesting that these mutations are selected for after exposure to TKIs [26].

Overexpression of certain receptors (e.g., HER2, c-Met), and their ligands, mediate TKI resistance by altering downstream PI3K and MAPK pathways in a way that promotes cancer cell proliferation/survival [27,28]. Exposure to TKI therapy has been associated with amplification of receptors and growth factors including insulin growth factor receptor (IGFR), fibroblast growth factor receptors (FGFRs), platelet-derived growth factor receptors (PDGFRs), and vascular endothelial growth factor (VEGF), all of which promote tumor cell proliferation and tumor blood supply [29]. The signal transduction pathways downstream of the EGFR receptor have significant interplay with a variety of other receptors. When the EGFR pathway is inhibited by TKIs, other signaling cascades are activated as an escape mechanism to bypass the inhibitory effects of the TKI and take advantage of the many other alternative signaling pathways.

Phenotypic transformation is another known mechanism by which EGFR-mutated NSCLC confers resistance to targeted therapy. Clinical investigations have demonstrated transformation of tumor cell populations from lung adenocarcinoma to squamous cell carcinoma or small cell lung cancer following treatment with targeted therapies [30,31]. Epithelial mesenchymal transition (EMT) is a process by which cells transform from epithelial to mesenchymal cells and is known to contribute to treatment resistance in many different types of tumors [32].

## 4. The Role of SBRT in Managing Metastatic NSCLC

Stereotactic body radiation therapy (SBRT) delivers focused, high-dose radiation to small tumor targets, typically in 3–5 fractions. Several randomized trials have defined the role for SBRT in the treatment of metastatic EGFR-mutated NSCLC, especially in those with limited metastatic disease burden [33,34]. Oligometastatic cancer describes a disease state in which the number and location of metastatic sites of disease are amenable to local therapy [35].

The SABR-COMET trial randomized patients with oligometastatic cancer (any histology and ≤5 sites of disease) to receive either SBRT to all sites of disease or systemic therapy alone. This trial demonstrated a progression free survival (PFS) benefit of SBRT to all sites of disease (median PFS 12 months vs. 6 months, p = 0.001) and an overall survival benefit (5 year OS 42% vs. 18%, p = 0.006) [36]. Thirty-six percent of patients treated on SABR-COMET had lung cancer, and standard-of-care systemic therapy was allowed on the trial at the discretion of the medical oncologist. 

A number of trials specifically designed for oligometastatic NSCLC have demonstrated a benefit to delivering SBRT to all sites of disease. The SINDAS trial is a phase III trial of newly diagnosed, oligometastatic EGFR-mutated NSCLC (≤5 oligometastases and no intracranial disease) randomized to receive TKI therapy alone or upfront SBRT to all sites of disease in addition to TKI therapy. The SINDAS trial reported a PFS benefit of upfront SBRT (median PFS of 12.5 months for TKI therapy alone vs. 20.2 months for upfront SBRT with TKI therapy, p < 0.001), as well as an OS benefit to upfront SBRT (median OS 17.4 months for TKI therapy alone vs. 25.5 months for SBRT and TKI therapy, p < 0.001) [33]. A phase II randomized study conducted by Gomez et al. randomized patients with NSCLC and 1–3 sites of metastasis, who received standard front-line systemic therapy for at least 3 months, to receive local consolidative therapy to metastatic disease with or without maintenance therapy compared to maintenance therapy alone. Surgery, radiation, or a combination of the two were considered local consolidative therapy options, although greater than 90% of patients on the trial received radiation treatment (48% of whom received SBRT). The Gomez trial reported both a statistically significant PFS and OS benefit to local therapy to sites of metastatic disease [37]. A phase II trial conducted by Iyengar et al. enrolled patients with NSCLC and ≤6 sites of metastatic disease following 4–6 cycles of first-line platinum-based chemotherapy with stable disease or partial response. Trial participants were randomized to receive either consolidative SBRT to all sites of disease followed by maintenance chemotherapy or maintenance chemotherapy alone. The trial arm having received consolidative SBRT was found to have superior PFS (median PFS 9.7 months vs. 3.5 months) [38]. There were no grade 5 toxicities on the SINDAS trial or on the trials conducted by Gomez et al. or Iyengar et al. 

## 5. Overcoming TKI Resistance: Preclinical Data

There are preclinical data to suggest radiation treatment as a potential strategy for overcoming TKI resistance in EGFR-mutated NSCLC. T790M mutations are known to be a main cause of TKI resistance in EGFR mutated NSCLC. Preclinical data have demonstrated that EGFR T790M mutations do not affect radiosensitivity [39]. However, when NSCLC cell lines harboring the EGFR T790M mutation were irradiated, the half maximal inhibitory concentration (IC50) of TKI therapy demonstrated a statistically significant decrease compared to the same cell line that did not receive radiation [39]. The results of this study suggest that ionizing radiation reduces TKI resistance.

Preclinical data also suggest that the combination of radiation therapy with targeted agents may provide an additional strategy for overcoming TKI resistance. An in vitro study found that inhibition of Heat Shock Protein 90 (Hsp90) via a targeted agent had a significant radiosensitizing effect on Gefinitib-resistant, EGFR-mutant NSCLC cell lines [40]. Another study demonstrated the radiosensitizing effect of dihydroartemisinin (DHA) on tumor tissues collected from patients with NSCLC [41].

## 6. SBRT as a Strategy for Overcoming Acquired TKI Resistance in the Setting of Oligoprogressive Disease

Cancer is defined as oligoprogressive when one or more sites of metastasis progress following an initial response to systemic therapy in the setting of otherwise controlled disease. Oligoprogressive disease is a distinct entity from oligometastatic disease. Patients in an oligoprogressive disease state have multiple tumor subclones, each possessing variable resistance to targeted therapy [42]. This explains why some metastatic lesions continue to respond to TKI therapy while others progress. The development of oligoprogressive disease further perpetuates drug resistance because populations of resistant tumor subclones have the ability to seed new metastases that will also exhibit drug resistance.

Some patients experience progression of one or more sites of metastatic disease while other sites of disease continue to remain stable or even respond to systemic therapy. In these cases, medical oncologists are faced with the decision to continue a patient on systemic therapy or switch to another systemic agent. Oncologists may be reluctant to switch therapies, especially in the case of a single progressing lesion in a background of well-controlled disease. There are retrospective data to suggest that patients who remain on targeted therapy following progression have better survival outcomes compared to those who are switched to another therapy at the time of progression, suggesting that subclones of their disease continue to be sensitive to targeted therapy [43,44].

Several studies have investigated the role of local therapy in oligoprogressive EGFR-mutant NSCLC with continuation of TKI therapy, several of which have specifically evaluated the role of SBRT in the oligoprogressive setting (Table 1). A retrospective study conducted at Memorial Sloan-Kettering Cancer Center evaluated local therapy (i.e., radiation therapy, surgical resection, or radiofrequency ablation) for patients with oligoprogressive EGFR-mutant NSCLC on TKI therapy. Most patients included in this study underwent surgical resection with only 17% of patients evaluated having received radiation treatment. Results demonstrated a trend toward longer time to progression (median PFS 10 months) when compared to patients who did not receive local therapy (p = 0.09). Median time to change of systemic therapy was 22 months and median OS was 41 months [45]. A similar study out of the University of Colorado retrospectively evaluated patients who received local therapy for oligoprogressive EGFR- or ALK-mutated NSCLC while on TKI therapy. 95% of oligoprogressive lesions included in this analysis were treated with radiotherapy, mostly using SBRT. Following local ablative therapy, patients were found to have a median PFS of 6.2 months [46]. There were no grade 3 or higher toxicities recorded for those having received extracranial local ablative therapy.

A single-arm phase II trial conducted by Iyengar et al. enrolled 24 patients with stage IV NSCLC (≤6 sites of extracranial disease) with disease progression on first-line systemic therapy. Trial participants received SBRT to all sites of disease concurrent with erlotinib. Results of the trial demonstrated a median PFS of 14.7 months and a median OS of 20.4 months [34]. EGFR testing was not mandatory and 0 out of 13 patients tested were found to be EGFR mutant.

A prospective study out of Duke University evaluated the effect of delivering SBRT to all sites of disease in patients with 1–5 metastatic cancer sites (of any primary tumor) and a life expectancy of at least 3 months [47]. Sixty-one patients were included in the study for a median follow up time of 20.9 months. PFS and OS were 33.3% and 81.5% at one year, respectively. At 2 years, PFS and OS were 22% and 56.7%, respectively. 27% of patients remained free of new metastases during follow up. 

**Table 1:** Trials evaluating SBRT as a strategy for overcoming acquired TKI resistance in EGFR-mutated NSCLC.

Author, Publication	Study Type	No. Patients	Inclusion Criteria	Study Arms or Intervention	Radiation Dose and Fractionation	Sequence of Targeted Therapy and Radiation	Results
Yu et al. [45]; PMID: 23407558	single institution retrospective	18	metastatic EGFR-mutant NSCLC with documented progression on EGFR TKI therapy	radiation therapy, radiofrequency ablation, or surgical treatment of a site of progressive disease	45–60 Gy in 3–5 fractions	Not reported	median time to progression after local therapy: 10 months median time until subsequent change in systemic therapy: 22 months median OS from local therapy: 41 months
Weickhardt et al. [46]; PMID: 23154552	single institution retrospective	25	metastatic ALK- or EGFR-mutant NSCLC treated with crizotinib or erlotinib with non-CNS progression and ≤4 sites of extra-CNS progression	local ablative therapy: SBRT, standard radiation therapy, or surgery	Median dose 40 Gy (range = 15–54 Gy)	targeted therapy withheld on days of local therapy and restarted the day following radiation, no change in dose	median PFS after local ablative therapy: 6.2 months
Santarpia et al. [48]; PMID: 32606174	single institution retrospective	36	advanced EGFR-mutant NSCLC with oligoprogression on first-line TKI therapy	hypofractionationed, high-dose radiation therapy (HD-HRT)	Median dose 30 Gy (range = 12–60 Gy)	Continuous delivery of targeted therapy throughout radiation treatment	median PFS after HD-HRT: 6.3 months median OS after HD-HRT: 38.7 months

Additionally, a retrospective study out of Italy evaluated the efficacy of “hypofractionated high-dose radiation therapy” in patients with oligoprogressive EGFR-mutated NSCLC following first-line TKI therapy. Results demonstrated a high rate of durable local control in irradiated sites of disease, with only 16.7% of patients going on to have a second progression [48]. Patients evaluated in this study continued on first-line TKI therapy and no grade 4 or higher toxicities were observed.

Therefore, one strategy to overcome resistance to targeted therapies in oligoprogressive disease is to use local therapy (either ablative or surgical) to treat resistant tumor subclones, which thereby prevents downstream seeding of additional drug-resistant metastases. Doing so may extend the duration of benefit of systemic therapy (thereby preserving subsequent-line options) and improve PFS. As discussed, prospective data on SBRT for oligoprogression are limited. Therefore, in these settings, a multi-disciplinary discussion is recommended and similar principals of radiation in the oligometastatic setting should be applied in the oligoprogressive situation in regards to maximum number of lesions felt to be reasonable to treat and radiation dose. 

### Ongoing Investigations of SBRT for Oligoprogressive EGFR-Mutant NSCLC

The role of SBRT in overcoming TKI resistance in EGFR-mutant NSCLC is the subject of several ongoing investigations (Table 2). A multicenter phase II trial out of the United Kingdom, known as the HALT trial (NCT03256981), is currently recruiting participants with stage IV EGFR-mutated NSCLC with oligoprogressive disease (defined as ≤3 sites of progressive disease) following an initial response to TKI therapy. Trial participants are randomized to receive SBRT to sites of oligoprogression in addition to TKI therapy or continued TKI therapy alone. The primary endpoint is PFS from time to SBRT to next progression.

A non-randomized pilot study sponsored by the National Cancer Institute is comparing upfront local ablative therapy followed by osimertinib to osimertinib followed by local ablative therapy followed by additional osimertinib (NCT02759835). Patients with no prior TKI therapy or with progressive disease after first- or second-generation TKI therapy that harbor the T790M mutation are allocated to the upfront osimertinib arm, whereas patients with disease progression on osimertitib are allocated to arm receiving local ablative therapy followed by osimertinib.

An open label, single arm study out of China is evaluating the combined use of pemetrexed, platinum-based therapy, bevacizumab, and durvalumab for 4–6 cycles followed by maintenance therapy with durvalumab +/− bevacizumab for patients with EGFR-mutant NSCLC that have progressed on first-line osimertinib (NCT04517526). Patients with disease progression on second-line therapy will be treated with salvage SBRT to sites of oligoprogression.

## 7. The Role of SRS in Treating EGFR-Mutated NSCLC with Acquired TKI Resistance

Survival outcomes for patients with EGFR-mutated NSCLC have improved over the past decade with the development of more effective systemic therapies. However, with longer survival times, the risk of developing brain metastases rises as well. Up to 70% of individuals with NSCLC develop intracranial metastases [49]. Brain metastases are more common among those with EGFR-mutated NSCLC compared to those with non-EGFR-mutated NSCLC [50]. The presence of brain metastases is associated with significantly worse clinical outcomes as well as significantly worse quality of life [51]. While first- and second-generation TKIs demonstrate poor penetrance of the blood-brain barrier, data have demonstrated the ability of third-generation TKI therapies to penetrate the blood-brain barrier and act on brain metastases [52]. As previously discussed in this review, however, resistance to TKI therapies including third-generation TKIs remains a problem. The concentration of TKI therapy in the brain is substantially lower compared to extracranial concentrations, so resistance to TKI therapy is a particular concern for progression of intracranial disease.

In the era of first- and second-generation TKI therapy, whole brain radiation therapy (WBRT) was suggested to be beneficial for brain metastases in EGFR-mutated NSCLC [53]. However, it is well established that WBRT is associated with cognitive decline and memory impairment [54,55]. With the development of third-generation TKIs that penetrate the blood-brain barrier, many oncologists consider upfront TKI therapy alone for individuals found to have intracranial metastases at the time of diagnosis with stage IV EGFR-mutated NSCLC. Several studies have demonstrated meaningful efficacy of Osimertinib against CNS metastases with intracranial response rates of 43–91%, depending on the number of brain metastases [56,57,58,59].

More recent studies have suggested a benefit of upfront stereotactic radiation therapy to brain metastases as a strategy for overcoming subsequent resistance to TKI therapy. A multi-institutional study comparing upfront SRS followed by TKI therapy, to WBRT followed by TKI therapy, to TKI therapy alone, for newly diagnosed EGFR-mutated NSCLC with brain metastases demonstrated superior outcomes with upfront SRS (median OS 46 months vs. 30 months vs. 25 months, respectively) [53]. This trial excluded patients having received prior TKI therapy including those demonstrating resistance to TKI therapy. 

A retrospective study of patients with EGFR-mutant NSCLC found that among those patients with disease progression on TKI therapy, 22% experienced their first progression in the CNS [46]. All of the patients with CNS progression included in this study received either SRS or WBRT for CNS disease progression. The median PFS in patients with CNS-only progression, treated with SRS or WBRT, was 7.1 months.

In summary, brain metastases should be addressed in patients with EGFR-positive NSCLC with a multidisciplinary approach including radiation oncology, medical oncology, and neurosurgery. SRS can be considered for patients with “limited” intracranial disease. For patients with targetable EGFR mutation and asymptomatic brain metastases potentially not amenable to SRS (i.e., numerous lesions likely requiring WBRT to control), the role of upfront radiation to brain metastases compared to salvage at the time of progression or symptoms remains to be defined in prospective trials.

### Ongoing Studies of SRS for Brain Metastases in EGFR-Mutant NSCLC

An ongoing multicenter phase II randomized trial out of Canada is comparing upfront Osimertinib in addition to SRS to brain metastases to Osimertinib alone for patients with treatment-naive EGFR-mutated NSCLC with brain metastases [60]. The primary endpoint for this trial is intracranial progression free survival, and secondary endpoints include intracranial overall response rate, time to whole brain radiation, time to SRS, rate of radionecrosis, OS, and neurocognitive function.

The OCEAN study is an ongoing phase II trial for patients with EGFR-mutated NSCLC and radiotherapy-naive CNS metastasis [61]. Patients enrolled on this trial will receive Osimertinib alone. The primary endpoint is the response rate of brain metastasis by PAREXEL criteria and secondary endpoints include PFS.

## 8. Strategies for Combining Targeted Therapy with Ablative Radiotherapy

The optimal timing of SBRT and TKI therapy remains unknown. There are preclinical data to suggest a synergistic effect of TKIs delivered concurrently with radiation therapy [62,63]. In the retrospective series reported by Santarpia et al. [48], which evaluated patients treated with continuous gefitinib therapy during hypofractionated high-dose radiation treatment, a majority of toxicities reported were grade 1–2, and no grade 4+ toxicities were reported. However, other reports have demonstrated an increased risk of severe radiation pneumonitis following concurrent delivery of TKI therapy and thoracic radiotherapy. A phase II study of upfront concurrent EGFR-TKI and thoracic radiotherapy for Stage IV EGFR-mutated NSCLC reported a grade 3+ radiation pneumonitis rate of 20% [64]. In a retrospective series reported by Jia et al. [65], among 11 patients treated with thoracic radiation concurrently with TKI therapy, six developed grade 3+ radiation pneumonitis including one case of grade 5 pneumonitis. These data suggest caution should be taken when planning for radiotherapy to the lung in a patient currently receiving TKI therapy, although additional prospective data is necessary to make conclusions regarding optimal timing of SBRT and TKI therapy. Given that the half life of most TKI therapies is less than 48 h, stopping TKI therapy 2–4 days prior to initiation of radiation therapy would be a reasonable strategy to avoid the risk of toxicity.

Regarding the timing of SRS to brain metastases and TKI therapy, several reports have demonstrated both the safety and feasibility of concurrent delivery of SRS and TKI therapy. One retrospective study reported a statistically significant improvement in intracranial progression free survival in those who received SRS concurrently with TKI therapy compared with sequential delivery of SRS and TKI therapy [66]. The same study found no adverse events associated with concurrent delivery of SRS and TKI therapy. A meta-analysis evaluating the safety and efficacy of concurrent TKI therapy with SRS or whole brain radiation therapy for brain metastases in the setting of Stage IV EGFR-mutated NSCLC reported a favorable toxicity profile and improved disease control rate [67].

## 9. Conclusions

There are clinical data supporting the continuation of patients on TKI therapy despite limited progression of one or more sites of metastatic disease in EGFR-mutated NSCLC. Based on the data reviewed here, the use of local ablative radiation techniques, including SBRT and SRS, to sites of disease progression is both safe and feasible, and results in durable local control of irradiated sites. This clinical strategy provides local control of TKI-resistant sites of disease, prevents TKI-resistant tumor subclones from seeding new tumors, and allows patients to continue on TKI therapy.

## Figures and Tables

**Table 2:** Ongoing studies evaluating SBRT for Oligoprogressive EGFR-mutant NSCLC.

NCT	Study Type	Inclusion Criteria	Study Arm(s)	Primary Endpoint
NCT03256981	phase II randomized multicenter study	advanced NSCLC with actionable mutation receiving targeted TKI therapy; ≤3 extracranial sites of progressive disease	arm 1: SBRT to oligoprogressive disease while continuing on TKI therapy arm 2: continued TKI therapy alone	PFS from time of randomization
NCT02759835	non-randomized, parallel assignment, open label trial	advanced lung adenocarcinoma with EGFR_sensitizing mutation with progressive disease after treatment with osimertinib who are eligible for local ablative therapy	local ablative therapy followed by osimertinib	PFS after treatment with local ablative therapy
NCT03410043	phase II randomized	NSCLC harboring EGFR T790M mutation that was acquired following progression on erlotinib, gefitinib, or afatinib	arm 1: osimertinib for 6–12 weeks then surgery or radiation therapy with continuation of osimertinib arm 2: osimertinib alone	PFS from start date of osimertinib
NCT04517526	multicenter, prospective, phase II	stage IV EGFR-mutant NSCLC with tumor progression following treatment with Osimertinib	pemetrexed + cisplatin/carboplatin + bevacizumab + duvalumab followed by bevacizumab and/or durvalumab maintenance therapy until progression; followed by stereotactic radiotherapy to oligoprogressive sites	PFS from start of study treatment

## Supplementary Materials

The following supporting information can be downloaded at: https://www.mdpi.com/article/10.3390/cancers14163983/s1, Supplementary File S1: Literature Search: Ablative Radiotherapy as a Strategy to Overcome TKI Resistance in EGFR-mutation NSCLC.
