# Supplementary material for: Ablative Radiotherapy as a Strategy to Overcome TKI Resistance in EGFR-Mutated NSCLC
Source: Cancers (Basel). 2022 Aug 18;14(16):3983. doi: 10.3390/cancers14163983 (PMC9406789; doi:10.3390/cancers14163983)
Supplement: Supplementary file 1 [file cancers-14-03983-s001.zip › cancers-1841612-supplementary.pdf]

Literature Search
Ablative Radiotherapy as a Strategy to Overcome TKI Resistance in EGFR-mutation NSCLC

Literature Search Performed on: April 11, 2022
Beginning Date: January 2010
End Date: March 2022
Database: Ovid MEDLINE(R) without Revisions <2010 to June Week 1 2021>
Search Strategy:
----
key words:
Lung neoplasms
Lung cancer
Lung carcinoma
Non-small cell lung cancer
NSCLC
Metastatic
Stage IV or Stage 4
Oligometastasis
Oligometastases
Oligometastatic
Oligopersistent
Oligoprogression
Oligoprogressive
Oligorecurrent
Polymetastatic
External beam radiation therapy or external beam radiotherapy
Stereotactic body radiation therapy or stereotactic body radiotherapy
SBRT
Stereotactic ablative radiation therapy or stereotactic ablative radiotherapy
SABR
Stereotactic radiosurgery
SRS
Radiotherapy
Chemotherapy
Targeted therapy
Epidermal growth factor receptor
EGFR
EGFR-mutant
Resistant
Refractory
Randomized
Randomised
Prospective

Criteria:
All adults (18 plus years)

Abstracts, review papers, and meta-analyses included
English language
2010 – current
Exclude case reports
